# Relationship Between Age and Weight Loss in Noom: Quasi-Experimental Study

**DOI:** 10.2196/18363

**Published:** 2020-06-04

**Authors:** Laura DeLuca, Tatiana Toro-Ramos, Andreas Michaelides, Elizabeth Seng, Charles Swencionis

**Affiliations:** 1 Ferkauf Graduate School of Psychology Yeshiva University Bronx, NY United States; 2 Seed Health Venice, CA United States

**Keywords:** older adults, DPP, mHealth, weight loss, lifestyle intervention, engagement

## Abstract

**Background:**

The prevalence of obesity and diabetes among middle-aged and older adults is on the rise, and with an increase in the world population of adults aged 60 years and older, the demand for health interventions across age groups is growing. Noom is an mHealth behavior change lifestyle intervention that provides users with tracking features for food and exercise logging and weighing-in as well as access to a virtual 1:1 behavior change coach, support group, and daily curriculum that includes diet-, exercise-, and psychology-based content. Limited research has observed the effect of age on a mobile health (mHealth) lifestyle intervention.

**Objective:**

The goal of the research was to analyze engagement of middle-aged and older adults using a mobile lifestyle or diabetes prevention intervention.

**Methods:**

A total of 14,767 adults (aged 35 to 85 years) received one of two curricula via an mHealth intervention in a quasi-experimental study: the Healthy Weight program (HW) by Noom (84%) or the Noom-developed Diabetes Prevention Program (DPP), recognized by the US Centers for Disease Control and Prevention (CDC). The main outcome measure was weight over time, observed at baseline and weeks 16 and 52.

**Results:**

Linear mixed modeling found age to be a significant predictor of weight at week 16 (*F*_2,1398.4_=9.20; *P*<.001; baseline vs week 16: *β*=–.12, 95% CI –0.18 to –0.07), suggesting that as age increases by 1 year, weight decreased by 0.12 kg. An interaction between engagement and age was also found at week 52 (*F*_1,14680.51_=6.70; *P*=.01) such that engagement was more strongly associated with weight for younger versus older adults (age × engagement: *β*=.02, 95% CI 0.01 to 0.04). HW users lost 6.24 (SD 6.73) kg or 5.2% of their body weight and DPP users lost 5.66 (SD 7.16) kg or 8.1% of their body weight at week 52, meeting the CDC standards for weight loss effects on health.

**Conclusions:**

Age and engagement are significant predictors of weight. Older adults lost more weight using an mHealth evidence-based lifestyle intervention compared with younger adults, despite their engagement. These preliminary findings suggest further clinical implications for adapting the program to older adults’ needs.

## Introduction

The prevalence of obesity among adults in the United States is on the rise, affecting nearly one half (40%) of adults aged 20 years and over, up 4% from 2014 [1,2]. Obesity is a known risk factor for insulin resistance associated with type 2 diabetes, placing individuals who are overweight or obese at risk for adverse health consequences [3]. Currently, 34.2 million Americans of all ages have diabetes; however, risk of diagnosis increases with age, with 26.8% of adults aged 65 years or older affected [4]. Type 2 diabetes remains the seventh leading cause of death for all ages, with increasing death rates faced by older adults (aged 55 to 74 years) [5].

Obesity-related conditions such as heart disease, stroke, and diabetes are among the leading causes of preventable early death, according to the US Centers for Disease Control and Prevention (CDC) [6]. With 960 million people aged 60 and over in the global population, anticipated to increase to 1.4 billion older adults by 2030 and to 2.1 billion by 2050, the prevalence of these chronic diseases is expected to rise further [7]. Evidence-based preventive measures and treatments that are feasible and effective for the growing older adult population could be used to counter these trends.

It is well established that adopting healthier lifestyle behaviors is essential to treating diabetes, prediabetes, and obesity [8]. Lifestyle interventions are a known effective approach in targeting weight reduction through dietary and exercise interventions and have been shown to reduce diabetes incidence [9]. Moderate (5% to 10%) weight loss interventions, including diet and exercise, have been shown to reduce mortality of older adults with obesity [10]. However, promoting weight loss in older adults can be controversial [11].

Research shows a potential risk for sarcopenic obesity that occurs when the loss of skeletal muscle mass from a weight loss intervention exacerbates sarcopenia, a condition of muscle atrophy which can be debilitating for an older person [12]. Further, certain epidemiologic studies suggest a protective effect of obesity in certain circumstances in older adults, known as the obesity paradox [3]. Criticisms of the paradox findings note older adults included in mortality studies likely represent a small portion of the population who did not already face fatal obesity-related complications earlier in life [13]. In many studies demonstrating the obesity paradox, distinctions between intentional versus unintentional weight loss were not made, so outcomes that indicate health risks from weight loss may largely be explained by illness-related weight loss [14]. Healthful weight loss is less likely to carry the same risks and can improve health outcomes.

The Diabetes Prevention Program (DPP) is an intensive lifestyle intervention shown to be cost-effective and successful in decreasing diabetes risk [15]. Promoting healthy weight loss is a central aspect of the DPP. Traditional group-based and face-to-face DPP lifestyle interventions have demonstrated efficacy to prevent diabetes in older adults. Employing diet and exercise lifestyle behavior changes reduced the incidence of diabetes by 71% in older adults. Older adults were more likely to reach 7% weight loss than younger adults (age 45 to 59 years [59%] vs age 25 to 44 years [48%]). At its 10-year follow-up, the DPP lifestyle intervention continued to show the greatest effect on diabetes incidence for the oldest participants (aged 60 to 85 years) compared with any other age group [16]. Program adherence may have played a role: session attendance was positively associated with age; adults aged 60 to 85 years participated in nearly twice as many sessions as younger adults [16]. Therefore, in-person DPP interventions are effective in tackling obesity, particularly for older adults.

With the widespread use of mobile health (mHealth) apps and broad availability of mHealth apps geared toward weight loss [17], there are increasing opportunities to implement evidence-based lifestyle and diabetes prevention interventions using mobile devices. Older adults have regular access to digital communications: 59% of adults aged 65 to 69 years, 49% of adults aged 70 to 74 years, and 31% of adults aged 75 to 79 years currently own smartphones, making mHealth interventions a viable option [18].

While some studies exploring technology-based DPP adaptations have included older adults, none to our knowledge have explored potential age effects on weight outcomes. One study explored the effectiveness of mHealth interventions specifically for this population and found 92% of participants completed at least half of the core DPP lessons and lost 7.5% of their body weight at the 12 month follow-up [19].

Clearly, mHealth interventions hold great promise as a cost-effective and feasible approach to weight loss for older adults. More information is needed to understand the specific utility of mHealth lifestyle interventions with consideration of potential age effects, as found in the original DPP program. One mHealth lifestyle program that has shown to be effective is Noom (Noom, Inc), with positive results found for overweight and prediabetic adults (aged 37 to 61 years) [20,21]. However, little is known about the impact of age on weight outcomes within this population.

This study sought to evaluate the role of age in predicting weight of participants of Noom’s Healthy Weight management (HW) and Noom’s Diabetes Prevention Program (DPP) over a short-term (16 week) and long-term (52 week) maintenance period. We hypothesized that older age would be associated with greater weight loss. A secondary aim was to evaluate the role of program engagement associated with age in predicting weight. We hypothesized older adults would be more engaged than younger adults which would predict greater weight loss.

## Methods

### Recruitment

Retrospective cohort data were extracted directly from Noom’s database in January 2019 and deidentified upon institutional review board approval from Albert Einstein College of Medicine. Noom is an mHealth behavior change lifestyle intervention that provides users with tracking features for food and exercise logging and weighing-in as well as access to a virtual 1:1 behavior change coach, support group, and daily curriculum that includes diet-, exercise-, and psychology-based content [20,21].

Participants were initially recruited by joining the Noom program in the app store (iTunes/Google Play). Informed consent to participate in research is provided by users during the initial sign-up for the program; users can choose to opt out of providing informed consent for research. Individuals in the HW program enrolled based on self-interest in weight loss and purchased the program for $129 for 4 months, on average. Individuals in the CDC-recognized DPP program, however, were encouraged to join following a prediabetes diagnosis from their health care provider and were offered the program for free through a health insurance offer. All Noom users are assigned a virtual health coach who successfully completed a CDC-recognized training course and are placed in a virtual group led by their coach. Users in both programs have access to the same features; the only difference that exists is the program curriculum users receive. While both programs focus on weight, healthy eating, and physical activity, the DPP program includes specific diabetes prevention content stemming from the CDC’s original DPP, which is not emphasized in the HW program.

**Figure 1:**
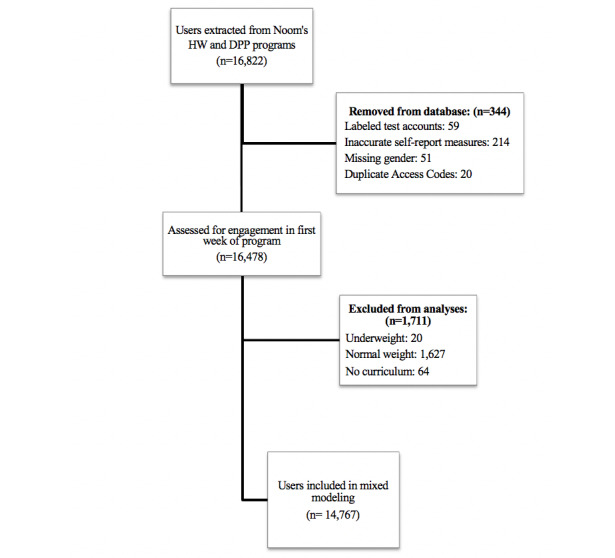
Description of data exclusion and inclusion of those who met study criteria.

Inclusion criteria were adults aged 35 years and older who began the HW or DPP program in June 2016 through January 2019 and had at least 1 program action within the first week of the program. The decision for selecting 35 years as the age minimum was made as a qualification of middle-aged adults whose degree of technology interaction was minimal during their youth. Users were considered ineligible and were excluded from the analyses if they self-reported a BMI categorized as underweight (<18 kg/m^2^) or normal weight (18.5 to 24.9 kg/m^2^; Figure 1) or were using the free version of Noom, as they do not have access to all program features (ie, no health coach, limited article content and tracking capabilities) and thus they did not receive the full intervention. Users were also excluded from analyses if they had inaccurate self-reported measures (as determined by large fluctuations in weight [ie, ±20 kg in 1 week]), test accounts (used by engineer developers at Noom to test the product), missing gender, and duplicate accounts (caused by errors with data extraction). Our study sample size is based on users who met the inclusion criteria.

### Measures

The primary outcome was self-reported weight, observed at baseline and weeks 16 and 52. To account for missing data at weeks of interest, 2-week ranges were observed around each time point and the mean of each range was used to calculate the final weight outcomes included in the analyses.

Engagement was observed in two ways. First, a definition was created to observe completion status. With the CDC’s DPP session attendance definition as a frame, it was decided to further adapt this previously used definition, originally created for in-person DPP programs, to improve applicability to mHealth. Therefore, program starters were considered as those who attended at least 1 session, defined as reading 1 article per week over 3 consecutive weeks or more during week 2 to week 6 and weighing in at least once per week for 2 weeks or more during week 2 to week 6. Program completers were considered to be those who read at least 14 articles (60%), a minimum of 1 per week, during the first 24 weeks of the core curriculum.

Engagement was also measured by users’ self-reported and behavioral-based program actions. Engagement variables included the number of self-reported meals logged, exercise logged, minutes of exercise logged, and frequency of weigh-ins, as well as behavioral-based steps recorded, articles completed (articles assigned divided by articles read), group interactions (group posts and comments), and messages to their individual coach, all tracked based on user program activity. The total value within each engagement variable was summed from baseline to week 52 and dichotomized (0 or 1); a score of 1 was given if a user logged at or above the 75th percentile cutoff for the individual variable. Composite scores for each user were calculated for all 9 engagement variables (score range of 0 = low engagement to 9 = high engagement).

### Statistical Analysis

Descriptive statistics were calculated for users’ baseline characteristics and expressed in means and standard deviations for continuous variables and frequencies and percentages for categorical variables (Table 1). Differences between demographics at baseline were observed using *t* tests for independent samples, chi-square analyses, and other nonparametric tests.

Linear mixed effects models evaluated changes in our primary outcome (weight). Linear mixed effects models estimate missing data within the analysis and are robust to data missing at random and not at random [22]. In our dataset, 2030 users of 14,676 recorded their weight at week 16 (±2 weeks) and 431 recorded their weight at week 52 (±2 weeks). Despite missing values and completion statuses, data from all users in the program were analyzed, and weight outcomes were predicted from the linear mixed models conducted.

Three analyses were completed. First, fixed effects were time and curriculum and their interaction to observe potential effects of curriculum. Second, age and time and their interactions were added, in addition to adjusting for curriculum, if found significant. Next, age, time, and total engagement and their interactions were included in the model. Time and the intercept for each participant were included as random effects in all models. Time was conceptualized as a 3-level categorical variable (week 0, 16, and 52). A first-order autoregressive covariance matrix yielded the best fit model for the repeated effect of time, using visual inspection and the Akaike information criteria. Significance tests were 2-sided with α set at .05. SPSS Statistics version 23 (IBM Corp) software was used to analyze the data.

**Table 1:** Descriptive statistics at baseline for participants of the Healthy Weight and Diabetes Prevention Program curricula.

Variable		HW^a^ curriculum (n=12,378)	DPP^a^ curriculum (n=2389)	*P* value
**Gender, n (%)**				.60
	Male	1451 (11.7)	289 (12.1)	—
	Female	10,927 (88.3)	2100 (87.9)	—
Age in years, median (IQR)		42.0 (38.0-47.0)	51.0 (44.0-58.0)	<.001
**Completion status, n (%)**				<.001
	Never engaged	9662 (78.1)	1480 (62.0)	—
	Engaged	372 (3.0)	60 (2.5)	—
	Starters	1767 (14.3)	458 (19.2)	—
	Completers	577 (4.7)	391 (16.4)	—
Initial weight (kg), mean (SD)		94.1 (20.4)	94.4 (20.5)	.53
Height (cm), mean (SD)		165.9 (7.1)	167.2 (9.5)	<.001
Baseline BMI (kg/m^2^), median (IQR)		32.6 (29.0-37.6)	32.2 (29.0-37.0)	.01

^a^HW: Healthy Weight program.

^a^DPP: Diabetes Prevention Program.

## Results

### Baseline Characteristics

Baseline characteristics are included in Table 1. Of the individuals selected at baseline from Noom’s database, 15.07% (2225/14,767) met criteria for starter in both the HW and DPP programs (Figure 1). Of the those who started both Noom programs, 43.51% (968/2225) of individuals completed the program (577/968 [59.6%] in HW and 391/968 [40.4%] in DPP). In the HW program, 88.27% (10,926/12,378) of participants were women, with a mean BMI of 32.6 (IQR 29.0 to 37.6) kg/m^2^. In the DPP program, 87.90% (2100/2389) of participants were women, with a median BMI of 32.2 (IQR 29.0 to 37.6) kg/m^2^.

DPP users were significantly older (median 51.0 [IQR 44.0 to 58.0] years) than HW users (median 42.0 [IQR 38.0 to 47.0] years; *P*<.001). Although the omnibus test suggested completion status differed between DPP and HW users (χ^2^_3_=520.93; n=14,767; *P*<.001), post hoc analyses yielded no significant differences with Bonferroni corrections (*P*<.006). DPP users were significantly taller (mean 167.2 [SD 9.5] cm) than HW users (mean 165.9 [SD 7.1] cm, t_14765_=–7.61; *P*<.001). HW users had significantly higher baseline BMI (median 32.6 [IQR 29.0 to 37.6] kg/m^2^) than DPP users (median 32.2 [IQR 29.0 to 37.0] kg/m^2^, *P*=.01). No other demographic characteristics significantly differed between curriculum groups (Table 1). The total sum of mean engagement variables for HW and DPP users across the study are found in Table 2.

Prior to running the mixed models, we observed weight loss throughout the program from users who provided data at week 16 and week 52 to better identify the amount of weight lost compared with CDC standards. Results showed that users who completed (as defined by our completer definition) the HW program lost on average 4.74 (SD 4.66) kg or 3.5% of their body weight at week 16 and 6.24 (SD 6.73) kg or 5.2% of their body weight at week 52. Users who completed the DPP program lost on average 5.61 (SD 8.06) kg or 5.7% of their body weight at week 16 and 5.66 (SD 7.16) kg or 8.1% of their body weight at week 52.

**Table 2:** Descriptive statistics of engagement variables from baseline to weeks 16 and 52.

Engagement measures		HW^a,b^ curriculum (n=2806), median, (IQR)		DPP^b,c^ curriculum (n=665), median (IQR)	
**Meals logged**					
	Week 16		88.0 (3.0-57.0)		195.0 (6.0-184.8)
	Week 52		91.0 (3.0-58.0)		218.0 (6.0-202.3)
**Articles completed**					
	Week 16		4.0 (0.3-2.4)		7.2 (0.4-6.0)
	Week 52		4.1 (0.3-2.4)		8.2 (0.4-6.7)
**Coach messages**					
	Week 16		26.0 (2.0-16.0)		29.0 (3.0-28.0)
	Week 52		26.5 (2.0-16.0)		35.0 (3.0-34.0)
**Steps tracked**					
	Week 16		228,923.0 (13,284.0-182,434.0)		331,572.0 (25,837.0-329,239.0)
	Week 52		267,270.5 (14,082.0-205,220.3)		629,241.0 (28,654.5-563,706.0)
**Weigh ins**					
	Week 16		10.0 (1.0-11.0)		20.0 (4.0-27.0)
	Week 52		10.0 (1.0-11.0)		31.0 (4.0-47.0)
**Exercises logged**					
	Week 16		13.0 (2.0-22.0)		25.0 (3.0-46.0)
	Week 52		13.0 (2.0-24.0)		33.0 (4.0-62.0)
**Minutes of exercise logged**					
	Week 16		227.5 (30.0-471.9)		735.0 (60.0-1332.5)
	Week 52		240.0 (30.0-490.0)		886.0 (60.0-1775.0)
**Group comments**					
	Week 16		9.0 (2.0-16.0)		11.0 (2.0-22.0)
	Week 52		9.0 (2.0-16.0)		13.0 (2.0-25.0)
**Group posts**					
	Week 16		5.0 (1.0-4.0)		9.0 (1.0-9.0)
	Week 52		5.0 (1.0-4.0)		9.0 (1.0-10.0)

^a^HW: Healthy Weight program.

^b^For participants who had engagement data available.

^c^DPP: Diabetes Prevention Program.

### Curriculum Effects

Tables 3 to 5 provide estimates and confidence intervals for the linear mixed effects models, with weight as the outcome. Results from the linear mixed model revealed that there was a significant interaction effect between curriculum groups and time (*F*_2,1401.0_=29.44; *P*<.001; Table 3). From baseline to week 16 and baseline to week 52, individuals in the DPP curriculum showed greater weight loss compared with HW users, losing 3.20 kg more at week 16 and 2.38 kg more at week 52 (baseline vs week 16: *β*=–3.20, 95% CI –4.02 to –2.37; baseline vs week 52: *β*=–2.38, 95% CI –4.17 to –0.59; Table 3). Therefore, the remainder of the models were adjusted for curriculum.

### Age Effects

**Figure 2:**
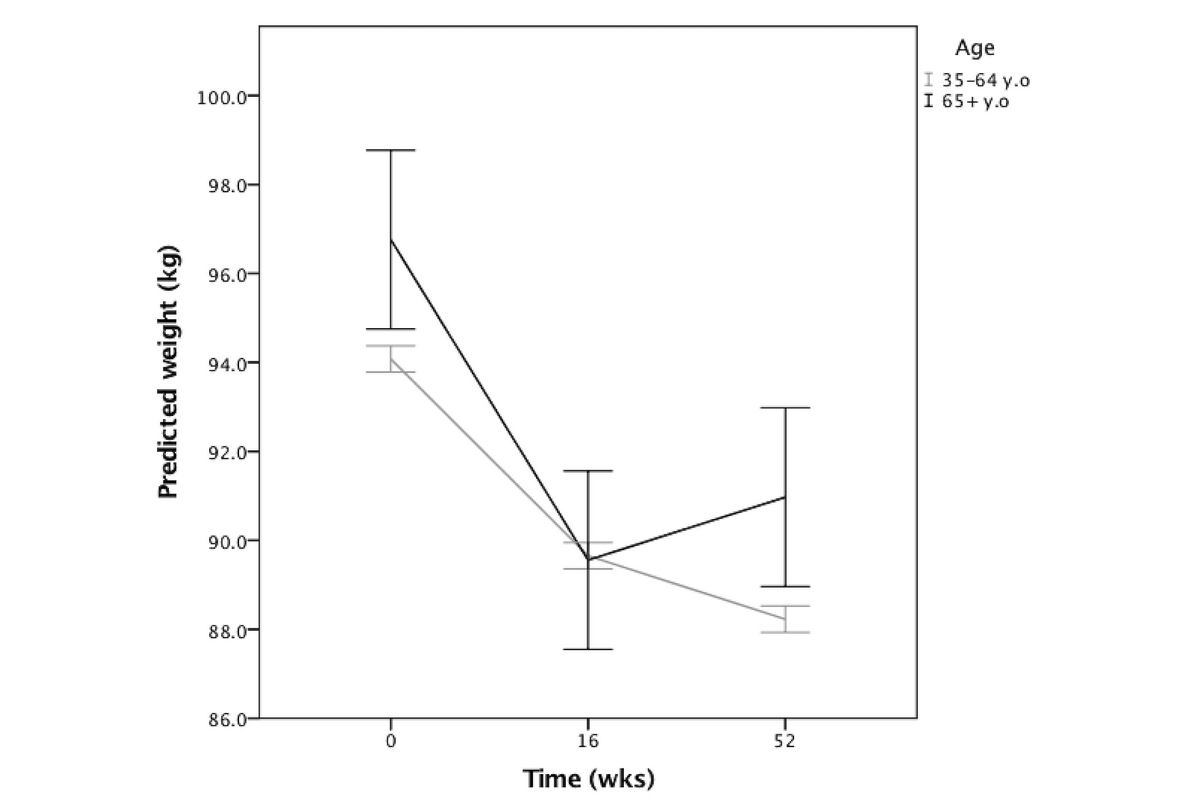
Interaction between age and time on predicted weight outcomes. Error bars: 95% CI. Data not distinguished by curriculum.

When we evaluated the effect of age, we found the interaction effect between age and time was significant (*F*_2,1398.4_=9.20; *P*<.001; Table 4). From baseline to week 16, adults who were older lost more weight earlier on compared with younger adults, such that for each additional year in age, weight decreased by an additional 0.11 kg (baseline vs week 16: *β*=–.11, 95% CI –0.16 to –0.06). However, from baseline to week 52, age was not a significant predictor of weight (baseline vs week 52: *β*=.003, 95% CI –0.11 to 0.11; Figure 2).

**Table 3:** Mixed model evaluating changes in weight by time and curriculum.

Effect	Estimate^a^	Standard error	*P* value
Intercept	94.09	0.18	<.001
DPP^b^	0.29	0.46	0.53
HW^c^	N/A^d^	N/A	N/A
Baseline	N/A	N/A	N/A
Week 16	–3.42	0.26	<.001
Week 52	–4.55	0.76	<.001
DPP*baseline^e^	N/A	N/A	N/A
DPP*week 16	–3.20	0.42	<.001
DPP*week 52	–2.38	0.91	0.01

^a^Estimate represents predicted value of weight.

^b^DPP: Diabetes Prevention Program.

^c^HW: Healthy Weight Program.

^d^N/A: Reference group used.

^e^*=interaction.

**Table 4:** Mixed model evaluating changes in weight by age and time.

Effect	Estimate^a^	Standard error	*P* value
Intercept	91.95	0.97	<.001
Age	0.05	0.02	.02
Baseline	N/A^b^	N/A	N/A
Week 16	0.5	1.24	.69
Week 52	–5.99	2.76	.03
Age*baseline^c^	N/A	N/A	N/A
Age*week 16	–0.11	0.03	<.001
Age*week 52	0.003	0.06	.96

^a^Estimate represents predicted value of weight.

^b^N/A: Reference group used.

^c^*=interaction.

**Table 5:** Mixed model evaluating changes in weight by age, engagement, and time.

Effect	Estimate^a^	Standard error	*P* value
Intercept	93.68	1.12	<.001
Age	0.02	0.02	.40
Baseline	N/A^b^	N/A	N/A
Week 16	1.79	1.31	.17
Week 52	–1.85	3.03	.54
Engagement	–1.33	0.39	<.001
Baseline*age^c^	N/A	N/A	N/A
Week 16*age	–0.12	0.03	<.001
Week 52*age	–0.02	0.06	.67
Age*engagement	0.02	0.01	.01
Baseline*engagement	N/A	N/A	N/A
Week 16*engagement	–0.13	0.07	.06
Week 52*engagement	–0.44	0.15	.004

^a^Estimate represents predicted value of weight.

^b^N/A: Reference group used.

^c^*=interaction.

### Engagement by Age and Time

**Figure 3:**
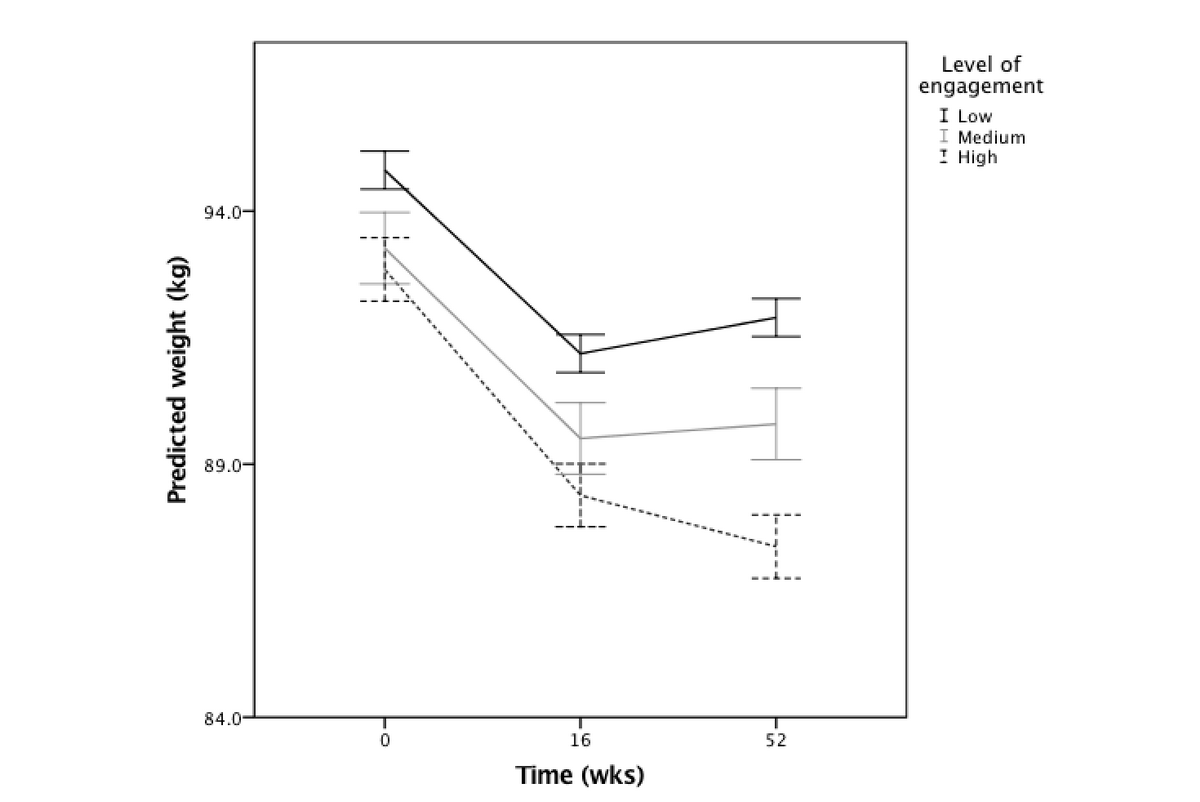
Interaction between time and engagement on predicted weight outcomes. Error bars: 95% CI. Data not distinguished by curriculum.

The last model evaluated interactions between age, time, and engagement, adjusting for curriculum. The 3-way interaction between age, engagement, and time was not significant and was removed from the model. Two-way interactions of engagement and time, age and time, and age and engagement were left in the model. A significant engagement effect (*F*_1,15238.5_=14.6; *P*<.001) was modified by the interactions between engagement and time (*F*_2,1368.7_=4.98; *P*=.01), age and engagement (*F*_1,14679.5_=6.70; *P*=.01), and age and time (*F*_2,1351.7_=10.37, *P*<.001; Table 5). In general, higher engagement was associated with lower weight over the entire study. At week 16, engagement was not yet significant as a predictor of weight (baseline vs week 16: *β*=–.13, 95% CI –0.27 to 0.00); at week 52, engagement was a significant predictor of weight such that as engagement increased by 1 composite score, weight decreased by 0.44 kg (baseline vs week 52: *β*=–.44, 95% CI –0.74 to –0.14; Figure 3).

The strength of the association between engagement and weight across the study differed by age; engagement was more strongly associated with weight for younger versus older adults (age and engagement interaction *β*=.02, 95% CI 0.01 to 0.04). Younger adults lost more weight when engaged; however, older adults lost weight over time despite their level of engagement.

As found in the prior model, age was associated with weight loss such that higher age was associated with greater weight loss at week 16 (*β*=–.12, 95% CI –0.18 to –0.07) but not at week 52 (*β*=–.02, 95% CI –0.14 to 0.09). Older adults lost more weight earlier on compared with younger adults such that for each additional year of age, weight decreased by an additional 0.12 kg.

## Discussion

### Principal Findings

This study explored the effect of age and engagement in predicting weight in a mobile intervention. To our knowledge, this is the first quasi-experimental study to consider age effects strictly in an mobile lifestyle intervention.

In support of our main hypothesis, higher age was associated with greater weight loss; older users lost more weight from baseline to week 16. Our second hypothesis that higher engagement would be associated with greater weight loss was supported, while our hypothesis that older users would engage more than younger adults was not found. Higher engagement was predictive of greater weight loss; however, the strength of the association differed by age. Although younger age was associated with engagement in predicting weight, older adults lost more weight from baseline to week 16 despite their level of engagement. These findings demonstrate that not only do older adults lose weight from mobile interventions, but they may benefit more compared with their younger counterparts.

### Comparison With Prior Work

mHealth interventions are used by older adults and appear to be an effective approach to weight loss. A meta-analysis by Valenzuela et al [23] of electronic health exercise programs for older adults (aged 67 to 86 years) yielded promising findings of technology as a well-accepted method, with the mean adherence as 91.3%. This is consistent with Svetkey et al [24], who found adults aged 60 years and older had both greater initial and sustained weight loss over 3 years compared with younger adults (aged younger than 50 years and between ages 51 and 60 years) in both counseling and internet-based intervention groups.

In our study, older age was not associated with engagement in predicting weight, which is not consistent with the DPP findings where adults aged 60 to 85 years attended nearly twice as much as adults aged 25 to 44 years [16]. Honas et al [25] found in a clinic-based weight loss program that younger adults were the only age group with an association with dropout (76% of individuals aged 51 to 60 years completed the program compared with 60% of participants aged 40 years and younger). A meta-analysis showed that in 13 studies, younger age was associated with higher attrition in weight loss interventions [26]. Additionally, adults aged 65 years and older were found to have higher self-monitoring rates and attend more sessions compared with younger adults (aged younger than 65 years) in an adapted DPP intervention [27]. One possible explanation for these findings is that older adults have a lack of work or family responsibilities (ie, fewer work demands). However, because we found program engagement mattered particularly for younger adults in our mobile intervention, these differences in findings compared with previous works may point to unique impacts of the use of technology, which younger adults likely have more experience with. Older adults experienced weight loss despite their total engagement, whereas younger adults benefited more from weight loss when they were engaged more with the program. It is likely that perception or presence of declining health may serve as a motivator for the aging population that extends beyond level of engagement to the mobile program. Further research should explore underlying motivators of engagement across age groups in mHealth interventions.

Our results showed that only 15% of users extracted from Noom met criteria for starters. One reason for this is that while we aimed to incorporate key engagement indicators, it is possible our definition may not capture true engagement within the program; thus, results may change with a different definition. Therefore, better mHealth definitions of engagement are needed. Dropout rates of 6% to 37% are common in mobile weight loss and diabetes interventions [28]; however, our high numbers particularly early in the program are likely related to a 2-week free trial period offered within the HW program at the time of extraction. More users may have joined who were not committed to long-term behavior change.

Throughout the 52 weeks, participants lost on average 6.24 (SD 6.73) kg or 5.2% of their body weight in the HW program and 5.66 (SD 7.16) kg or 8.1% of their body weight in the DPP program. These results meet the CDC standards that state that individuals who lose 5% of body weight or more can benefit from reduced risk for chronic diseases related to obesity [29]. Further research is needed to explore the feasibility of participants’ experience with technology interventions to better understand potential barriers that may exist. Scheibe et al [30] showed that older adults reported difficulty in understanding the functionality of the apps’ touch sensitive areas and that the visual representations were too small to be easily visible as reasons against using mobile diabetes interventions. As findings did not show a strong interaction of age and engagement for older adults, it is likely that barriers exist that affect the overall feasibility of the mobile intervention, requiring adaptations to enhance the users’ experience.

### Limitations

Participants were self-selecting and results may not generalize to populations with less intrinsic interest in weight loss. As our study is observational, the effect of the intervention against a control group is unknown. We decided to use initial weight versus first weigh-in as our baseline weight, given missing data concerns. It is likely the initial weight input at the time of sign-up may not reflect a true weight on a scale, as it is hypothesized many users estimate how much they believe they weigh during the sign-up phase. Third, completion status criteria for never engaged was determined based on overall engagement in week 1. Therefore, participants who were excluded may have engaged in later weeks. Additionally, as some forms of engagement included self-reports, it is hard to distinguish if a lack of exercise logged reflects a lack of exercise versus a lack of reporting. Therefore, behavioral-based engagement steps recorded are more likely to indicate a true level of engagement. Fourth, potential bias in motivational differences likely exists between users in HW and DPP, as users paid for the HW program versus users who received the DPP program for free.

Because of the retrospective design, it was not possible to assess whether users had a prediabetes or diabetes diagnosis in the HW program. The HW program is available to anyone who is able to afford it and owns a smartphone; thus, users may have additional underlying health conditions that were unknown. Finally, as mentioned previously, it is likely the CDC’s definition of attendance does not directly apply to mHealth interventions and may not have optimally captured the true findings of dropout rate or completers of the program.

### Conclusions

In conclusion, age and engagement appear to play a significant role in predicting weight while using a mHealth lifestyle intervention at weeks 16 and week 52 in this study. Not only did older adults lose more weight from baseline to week 16, but they may benefit more compared with younger adults. Further analyses are needed to explore potential age differences to better optimize older adults’ experience within a mobile intervention.

## Abbreviations

CDC: Centers for Disease Control and Prevention
DPP: Diabetes Prevention Program
HW: Healthy Weight
mHealth: mobile health
